# A Bioinspired Flexible Pressure Sensor with High Linearity Based on a Three-Dimensional Porous Structure

**DOI:** 10.3390/biomimetics11060376

**Published:** 2026-05-29

**Authors:** Xingze Chen, Xin Wang, Huansheng Wu, Cong Wang, Yonghua Wang, Linpeng Liu, Ji’an Duan

**Affiliations:** 1 State Key Laboratory of Precision Manufacturing for Extreme Service Performance, College of Mechanical and Electrical Engineering, Central South University, Changsha 410083, China; 253711063@csu.edu.cn (X.C.); 253711004@csu.edu.cn (X.W.); 253703011@csu.edu.cn (H.W.); wangcong@csu.edu.cn (C.W.); duanjian@csu.edu.cn (J.D.); 2 Ministry of Education Key Laboratory for Cross-Scale Micro and Nano Manufacturing, Changchun University of Science and Technology, Changchun 130022, China; yonghua@cust.edu.cn

**Keywords:** pressure sensor, high linearity, porous sensor, human motion monitoring

## Abstract

Flexible pressure sensors with a porous architecture are highly desirable for wearable health monitoring and intelligent human–machine interaction, owing to their excellent comfort and conformability to human motion. However, conventional porous sensors often suffer from poor signal accuracy and unstable output, which limit their capability for precision sensing. To address these challenges, we designed and fabricated a flexible pressure sensor with exceptional linearity by mimicking the unique surface structure of Iron Cross Begonia (*Begonia masoniana*) leaves. The sensor is constructed using a readily available melamine foam as the backbone: a porous sensing scaffold is first obtained via a simple dip-coating process, and a film featuring bioinspired protrusions is fabricated by repeated replica molding. Lamination of these two components yields a stacked sensor device. Characterization demonstrates that the sensor achieves a broad pressure detection range of up to 350 kPa, with a minimum resolvable pressure of 250 Pa, and exhibits an excellent linearity of 0.999 over its entire working range (0–350 kPa). Moreover, the sensor shows stable responses under varying loading frequencies, is capable of detecting low-frequency signals, and retains its performance without notable degradation even after 5000 repeated loading-unloading cycles. In practical applications, the sensor accurately monitors flexion and extension movements of the wrist, finger, neck, and knee, capturing human motion signals with high fidelity. Furthermore, it enables information encoding and transmission through finger gestures. The proposed bioinspired structural design strategy effectively enhances the overall performance of porous pressure sensors, offering a new paradigm for the development of flexible sensing devices with promising applications in wearable health monitoring, human motion detection, and human–machine interaction.

## 1. Introduction

As a critical category of mechanical sensors, pressure sensors are widely used in pressure monitoring, biomedical engineering, and human–machine interaction [1,2,3,4,5,6]. In recent years, flexible pressure sensors have garnered extensive attention in wearable health monitoring, human motion capture, human–machine interaction, smart healthcare, and flexible electronics, owing to their lightweight nature, bendability, conformability to the human body, and real-time response [7,8,9,10,11,12,13]. Compared with traditional silicon-based counterparts, they also offer high sensitivity and low manufacturing cost. Most innovative flexible sensors have found applications in flexible electronic skin, health monitoring, and human–machine interaction [14,15]. In general, the working mechanisms of flexible pressure sensors fall into three categories: piezoresistive [16,17], piezoelectric [18,19], and capacitive [20,21]. Among these, piezoresistive sensors are widely adopted in device design due to their excellent sensitivity and linearity to pressure and deformation, as well as their simple working principle and structural design. The fundamental sensing mechanism of a piezoresistive pressure sensor lies in the fact that when an external pressure is applied to the sensor, the microstructure of the sensitive material deforms, leading to a change in its internal conductive pathways. This consequently alters the overall resistance (or resistivity) of the device, and the pressure information can be indirectly obtained by detecting the resistance change. In particular, for piezoresistive sensors based on conductive composites, the conduction mechanism is commonly explained by the “percolation theory” and the “tunneling effect”. In the absence of external pressure, the conductive fillers are dispersed in the insulating polymer matrix. A certain distance exists between adjacent filler particles, and the conductive network is partially disconnected, resulting in a relatively high initial resistance of the device. When pressure is applied, the polymer matrix undergoes compressive deformation, reducing the distance between filler particles. On one hand, the number of direct contact conductive pathways increases (percolation effect); on the other hand, when the interparticle distance decreases to the nanometer scale, electrons can cross the potential barrier via the tunneling effect. The synergistic action of these two mechanisms leads to a significant decrease in the resistance of the sensor under applied pressure. For instance, when an external load is applied, the sensor converts mechanical deformation into a change in internal resistance, which is further detected through variations in current or voltage.

It is well established that critical performance metrics-such as sensitivity, linearity, operating range, and minimum force resolution-determine the overall quality of a sensor. To enhance the comprehensive performance of sensors, microstructural design inside the device has proven to be an efficient and viable strategy, as demonstrated by groove structures, microcracks, and micropillar arrays [22,23,24,25,26]. Meanwhile, porous materials, featuring high specific surface area and strong deformability under compression, are often selected as ideal substrate candidates for fabricating wide-range flexible pressure sensors [27,28,29,30,31,32,33,34]. However, when porous foam materials are used, the contact between the foam surface and the electrode is typically point-contact. Under repeated external loading, the sensor often operates in a contact-non-contact regime, leading to unstable signals and inevitable white noise, an undesirable effect. This indicates that relying solely on porous structures to fabricate flexible pressure sensors with a wide range and high linearity still suffers from signal instability, poor response linearity, and long-term signal drift, making it difficult to meet the demands of practical mechanical engineering applications. Therefore, developing pressure sensors that simultaneously offer high signal fidelity, high linearity, and a broad operating range remains a critical research direction in flexible electronics.

To improve the overall performance of sensors, surface microstructure engineering has been demonstrated as an effective and efficient strategy. Unique microstructures on natural biological surfaces enable highly sensitive detection of external weak stimuli through mechanisms such as stress concentration, graded contact, and synergistic deformation, providing rich inspiration for sensor structure optimization [35,36,37,38,39,40,41]. Over the past few years, researchers have developed sensing interfaces with protrusions, wrinkles, arrays and other morphologies by mimicking biological microstructures from plant leaves, insect cuticles, etc., effectively enhancing sensor sensitivity and linearity [42,43]. However, most studies have focused on single-sided microstructures, and there remain deficiencies in the synergistic improvement of pressure range, dynamic stability, low-frequency detection, and long-term reliability. Moreover, complex fabrication processes and high costs limit their practical deployment.

Through this investigation, it has been found that the leaves of *Begonia masoniana*, commonly known as Iron Cross Begonia, are covered with densely distributed, rigid protuberances that exhibit a highly regular morphological arrangement across the entire leaf surface. Under force, these protrusions exhibit stress concentration and graded deformation, enabling an inherently efficient response to weak external forces. Inspired by this, we propose a flexible pressure sensor design strategy that integrates a double-sided bioinspired microstructure with a porous conductive sponge. Using a simple template method, we fabricate a conductive film with a biomimetic protrusion array. This film is then assembled with a carbon black-modified melamine sponge into a stacked structure of MF-CSP-MF. By leveraging the dual sensing mechanisms of enhanced surface contact and internal deformation of the porous sponge, we simultaneously improve the operating range, sensitivity, linearity, dynamic response, and stability. Systematic characterization shows that the sensor exhibits excellent linear response (R^2^ = 0.999) over a wide pressure range (0–350 kPa), fast response and recovery times, and the ability to resolve small pressure changes. The signal remains stable across a frequency range of 0.5–3.0 Hz, and the sensor can detect weak dynamic signals as low as 0.05 Hz. No significant performance degradation is observed after 5000 cyclic loading-unloading tests. In practical applications, the sensor can accurately monitor joint movements of the wrist, finger, neck, knee, elbow and other body parts, and can also output Morse code signals through finger gestures, demonstrating its potential for human motion monitoring, health assessment, and human–machine interaction.

## 2. Materials and Methods

### 2.1. Materials

Commercial conductive carbon ink was purchased from M&G Chenguang Stationery Co., Ltd., (Shanghai, China). Polyurethane sponge (100 ppi) with a height of 10 mm was bought from Kunshan Shangte New Material Co., Ltd., (Suzhou, China). Multi-walled carbon nanotubes (MWCNTs) were obtained from Suzhou Tanfeng Graphene Technology Co., Ltd., (Suzhou, China). Waterborne polyurethane dispersion was supplied by Zhengmeisu Adhesive Co., Ltd., (Guangzhou, China). Sandpaper (60-grit) was purchased from Hubei Yuli Abrasive Belt Group, (Xianning, China). Melamine foam was acquired from a local market.

### 2.2. Preparation of Sensors

**Figure 1:**
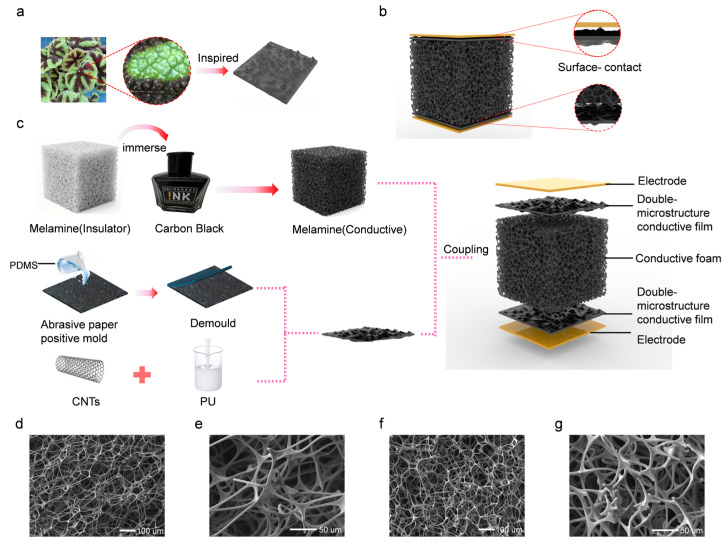
Fabrication of a highly sensitive pressure sensor bioinspired by Iron Cross Begonia (*Begonia masoniana*). (**a**) Bioinspired design workflow. (**b**) Assembly diagram of the sensor components. (**c**) Sensor fabrication process flow. (**d**) SEM image of the pristine sponge (before immersion) at 150× magnification; (**e**) SEM image of the pristine sponge (before immersion) at 150× magnification; (**f**) SEM image of the sponge after immersion in carbon ink at 150× magnification; (**g**) SEM image of the sponge before immersion in carbon ink at 150× magnification.

Inspired by the natural microstructure on the surface of Iron Cross Begonia (*Begonia masoniana*) leaves, this paper reports the design and fabrication of a highly sensitive flexible pressure sensor based on a dual-microstructure interface. As shown in Figure 1a, the leaf surface of *Begonia masoniana* is covered with numerous microscopic hard protrusions, which enable efficient response to weak external forces through stress concentration and graded deformation. Drawing on this biological concept, we constructed a conductive film featuring a bioinspired protrusion array and integrated it with a conductive sponge to form a stacked sensing architecture of MF-CSP-MF. This architecture provides the core structural support for the sensor’s high sensitivity. The detailed fabrication process is illustrated in Figure 1c. First, an insulating melamine sponge was immersed in carbon black ink and subjected to ultrasonic treatment for 2 h to ensure that the ink fully infiltrated the sponge skeleton. The sponge was then dried in an oven at 70 °C for 2 h, yielding a conductive sponge that retained an intact porous structure. The sponge was then dried in an oven at 70 °C for 2 h, yielding a conductive sponge that retained an intact porous structure. From Figure 1d–g, we can observe that after immersion in carbon ink, more carbon atoms are attached to the sponge skeleton, which greatly enhances its electrical conductivity. This is also strongly supported by the energy-dispersive X-ray spectroscopy (EDS) spectra obtained from scanning electron microscopy. For the preparation of the double-sided microstructured conductive film, sandpaper (60-grit) was used as a microstructure template. PDMS prepolymer was coated onto the sandpaper surface, cured, and demolded to obtain a PDMS inverse template. Subsequently, a mixed suspension of multi-walled carbon nanotubes (MWCNTs) and polyurethane (PU) was cast onto the PDMS template. After curing and demolding, a single-sided microstructured conductive film was obtained. Repeating this casting process and curing the two single-sided films together produced a double-sided microstructured conductive film. Finally, the double-sided microstructured conductive film, the conductive sponge, and copper electrodes were assembled in sequence. The interfaces were bonded and cured using the same CNTs/PU mixed suspension together with silver paste, completing the electrode encapsulation and yielding the complete flexible pressure sensor.

### 2.3. Characterization and Testing

The surface microstructure morphology of the porous sensor was carefully observed using a super-depth-of-field microscope (VH-5000, Keyence, Foshan Zongben Optical Instrument Co., Ltd., Guangzhou, China). The basic performance test was conducted by applying pressure to the sensor using an electrically linear workbench with positioning control. The resistance of the pressure sensor was measured in real-time using a multimeter (DMM6500, Agilent, Tektronix Technology Co., Ltd., Shanghai, China). The mechanical force applied to the pressure sensor was measured using a strain gauge (HP-1kN, Handpi in the instrument, Handpi Instrument Co., Ltd., Yueqing, China).

## 3. Results and Discussion

### 3.1. Electromechanical Response of the Sensor

**Figure 2:**
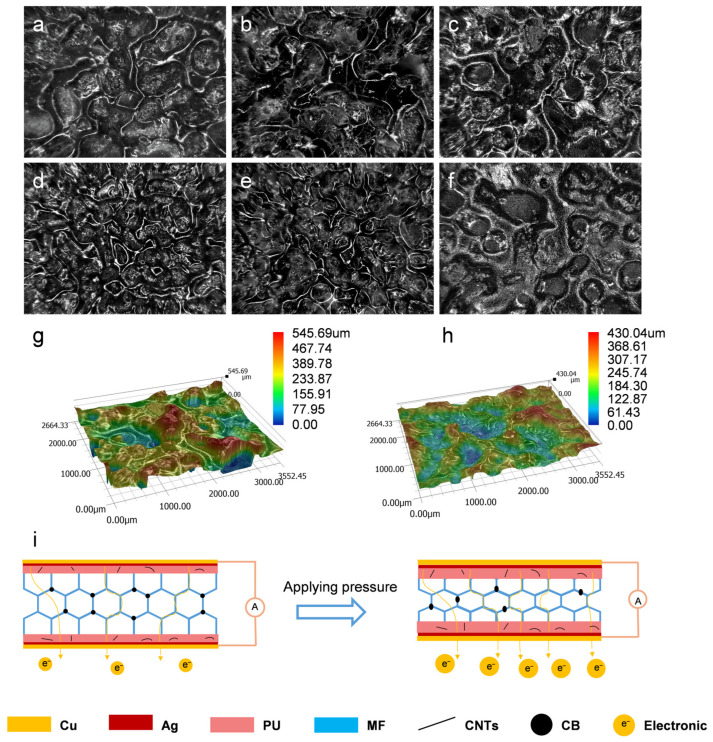
Surface morphology and sensing mechanism of the sensor. (**a**–**c**) Digital microscope images of the 60-mesh sandpaper template (**a**), the PDMS replica obtained after a single molding step (PDMS base/curing agent ratio = 10:1) (**b**), and the resulting microstructured conductive film (**c**). (**d**–**f**) Digital microscope images of the 100-mesh sandpaper template (**d**), the corresponding PDMS replica (10:1) (**e**), and the microstructured conductive film (**f**). (**g**) Three-dimensional profilometric image of the microstructured film obtained by secondary replica molding using the 60-mesh sandpaper template. (**h**) Three-dimensional profilometric image of the microstructured film obtained by secondary replica molding using the 100-mesh sandpaper template. (**i**) Schematic illustration of the sensing mechanism of the pressure sensor.

The bioinspired microstructured surface fabricated by replica molding is characterized in Figure 2. Specifically, Figure 2a–f show the 60-mesh and 100-mesh sandpaper templates, the corresponding PDMS replicas obtained after a single molding step, and magnified views of the resulting conductive film featuring well-defined microprotrusion structures. The secondary replica exhibits a surface microstructure that closely matches that of the 60-mesh sandpaper, confirming the successful replication of the microstructures. Figure 2g,h present three-dimensional profilometric images, revealing the surface topography of the microstructured conductive film. Figure 2i illustrates the deformation behavior of the sensor under applied pressure.

When compressed, the internal porous sponge structure deforms, leading to a reduction in the number of conductive pathways while simultaneously increasing alternative conduction routes. As a result, the overall resistance of the device decreases. Moreover, during the gradual loading of external pressure, the bionic protrusion structure exhibits distinct graded and progressive deformation characteristics. Under low-pressure loads, stress concentration first occurs at the tips of the protrusions, which undergo preferential elastic deformation. This mechanism endows the sensor with excellent sensitivity to low-pressure stimuli. As the external load continues to increase, the microprotrusions as a whole become progressively compacted, and subsequently, the intermediate porous sponge matrix takes over the load-bearing role. Throughout the entire force-bearing process, the effective contact area of the sensor evolves continuously and linearly with the applied external pressure. This behavior effectively avoids the common drawbacks associated with conventional smooth-film composite structures, such as premature compaction, rapid response saturation, and a narrow linear sensing range.

This advantageous behavior can be further understood by comparing the bionic micro-protruded surface with a conventional flat structure. In the flat structure without micro-protrusions, the film fits intimately with the sponge over the entire contact area, which prevents load dispersion and leads to pronounced local stress concentration. Such a configuration is rigid under low pressure and hardly undergoes micro-deformation. A slight increase in pressure causes an abrupt collapse of the sponge pores and an instantaneous stress release, quickly driving the structure into compaction saturation. Consequently, the discontinuous deformation and severe stress fluctuations result in poor linearity and early saturation. In contrast, for the bionic structure with arrayed micro-protrusions, the overall load is shared among numerous independent protrusion units. The load is evenly distributed without obvious local stress concentration. Under low pressure, the protrusion tips deform preferentially, enabling high sensitivity to weak pressure. As the pressure increases, the contact state transitions smoothly from isolated protrusion contact to gradual overall compaction. The entire deformation process remains stable with uniform stress release, avoiding abrupt changes and early saturation, thereby achieving a wide linear response range.

**Figure 3:**
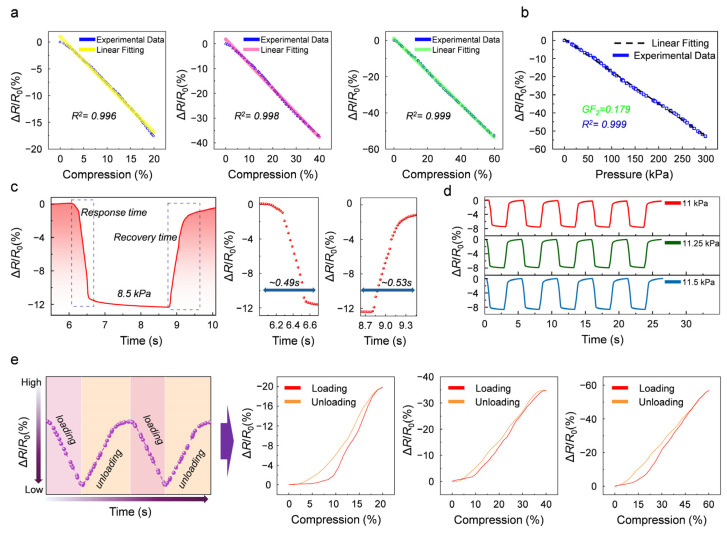
Performance of the sponge pressure sensor. (**a**) Relative resistance change at compression ratios of 20%, 40% and 60%. (**b**) Resistance change over a pressure range of 300 kPa. (**c**) Response and recovery times under 8.5 kPa load. (**d**) Relative resistance change under small incremental pressure steps. (**e**) Real-time relative resistance during loading and unloading cycles at 8.5, 22.5 and 52.5 kPa.

First, to evaluate the response of the sensor over its strain range, systematic measurements were performed at different compression levels. As shown in Figure 3a, polynomial fitting was applied to three representative compressive strain intervals: 20%, 40% and 60%. The corresponding coefficients of determination (R^2^) were 0.996, 0.998 and 0.999, respectively, indicating that the sensor exhibits consistent resistance strain behavior within these compressive strain regions. Even when tested up to a compressive strain of 60%, the device maintained a high fitting accuracy (R^2^ = 0.999), demonstrating stable and reliable output under large compressive deformation. Such excellent linearity across a wide strain range is rarely achieved in porous structured piezoresistive sensors, highlighting the advantage of the present design.

To further characterize the sensitivity of the sensor, a compressive stress of 300 kPa was applied, and the relative resistance change is shown in Figure 3b. Linear fitting of the data yielded a gauge factor of 0.179 with a correlation coefficient R^2^ = 0.996, confirming excellent linearity and stable response characteristics that fully meet the requirements for high precision detection. Compared with previously reported pressure sensors, our sensor achieves significantly higher linearity [27,28,30,32,39]. It is well known that response time and recovery time are important sensing parameters for pressure sensors, determined primarily by the composition of the conductive material and the sensor architecture. Subsequently, a pressure of 8.5 kPa was applied to the sensor. As shown in Figure 3c, the response time was approximately 0.49 s and the recovery time was approximately 0.53 s, indicating a fast signal response speed. These values compare favorably with many previously reported flexible pressure sensors, confirming the suitability of our device for real-time monitoring applications.

As demonstrated in Figure 3d, when a small incremental change in applied pressure (from 11 kPa to 11.25 kPa and from 11.25 kPa to 11.50 kPa) was introduced under an applied pressure of 11 kPa, the relative resistance change exhibited a pronounced variation, indicating that the sensor has a distinct response to subtle pressure changes. This high resolution is essential for applications requiring the detection of slight pressure variations, such as pulse wave monitoring or delicate tactile sensing. Furthermore, to investigate hysteresis, one loading–unloading cycle was arbitrarily selected from cyclic tests at applied pressures of 8.5 kPa, 22.5 kPa and 52.5 kPa to evaluate the unloading response and recovery of the sensor. The relative resistance change curves for the unloading (recovery) versus loading (compression) cycles are shown in Figure 3e. The loading and unloading profiles are in close agreement, indicating that the device does not suffer from significant signal hysteresis. This low hysteresis behavior ensures measurement accuracy and repeatability, which are critical for long-term wearable applications.

**Figure 4:**
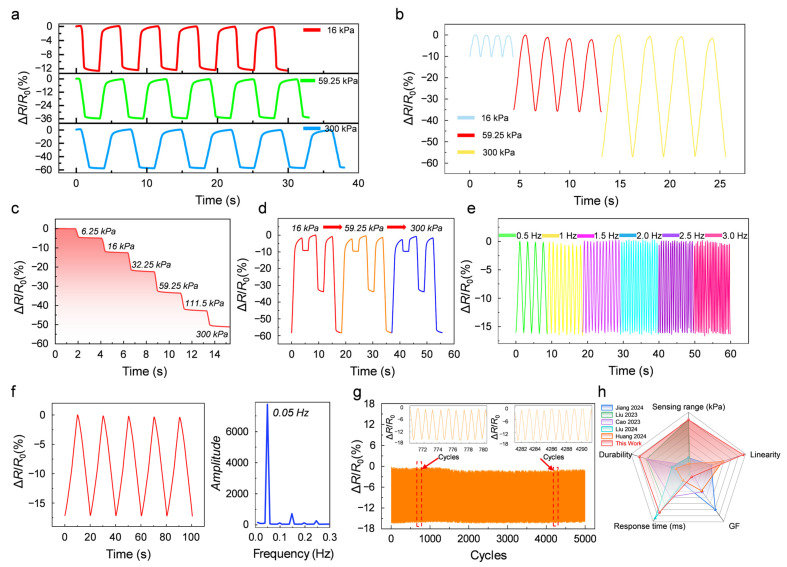
Dynamic response of the sponge pressure sensor. (**a**) Response under cyclic dynamic and static loads at 16, 59.25 and 300 kPa (peak pressure duration: 5 s). (**b**) Relative resistance change under cyclic dynamic loads of 16, 59.25 and 300 kPa. (**c**) Relative resistance change under stepwise pressure from 6.25 to 300 kPa. (**d**) Relative resistance change under cyclic step-pressure loading. (**e**) Response at constant amplitude but varying frequencies (0.5–3.0 Hz). (**f**) Resistance change at 0.05 Hz and corresponding FFT spectrum. (**g**) Stability over 5000 loading-unloading cycles at fixed pressure. (**h**) Comparison of our pressure sensor with other reported pressure sensors in terms of working range, linearity, sensitivity, response time, and durability [26,27,29,33,34].

Next, to systematically evaluate the dynamic response of the fabricated pressure sensor, we first applied cyclic pressure loads with different amplitudes (16 kPa, 59.25 kPa, and 300 kPa), each maintained for a duration of 5 s, as depicted in Figure 4a. The sensor exhibited highly reproducible response curves during the loading-hold-unloading process: the peak response increased regularly with the applied pressure, and the signal remained stable without noticeable drift under constant pressure, demonstrating excellent response stability and linearity. Figure 4b presents the response when we subsequently removed the pressure hold phase and applied cyclic dynamic pressures at the same amplitudes (16 kPa, 59.25 kPa, and 300 kPa). The relative resistance change in the sensor rose and fell rapidly and synchronously with pressure application and release, and the response amplitude was positively correlated with the pressure magnitude. This trend was fully consistent with the static sensitivity measurements presented in Figure 3b.

Furthermore, the sensor response to stepwise pressure loading was characterized under sequentially increasing step pressures ranging from 6.25 kPa to 300 kPa. Figure 4c presents the results, where the response signal exhibited a clear stepwise decrease, and the response level at each pressure plateau remained stable without significant relaxation. Subsequent cyclic step-pressure tests (16 kPa to 59.25 kPa, and 59.25 kPa to 300 kPa) are displayed in Figure 4d, revealing fast and stable step responses. The peak response at each pressure stage decreased regularly with increasing pressure, demonstrating good step-discrimination capability.

To evaluate the frequency-response characteristics, cyclic pressure loads at frequencies ranging from 0.5 Hz to 3.0 Hz were applied under a fixed pressure amplitude. Figure 4e illustrates the resulting response signals. The sensor produced stable and consistent response curves across the entire tested frequency range, with no noticeable attenuation in the relative resistance change amplitude, indicating excellent signal fidelity and frequency independence within this bandwidth. To further explore the low-frequency detection capability, we tested the sensor’s response under a low-frequency cyclic pressure of 0.05 Hz. Figure 4e (inset or continuation) demonstrates that the sensor outputs a clear and stable periodic response waveform even under such low-frequency excitation. The corresponding fast Fourier transform (FFT) analysis showed a dominant signal peak at 0.05 Hz without significant spurious components, confirming that the sensor can reliably detect weak dynamic pressures as low as 0.05 Hz.

Finally, we conducted a long-term cyclic stability test of the sensor for 5000 consecutive loading-unloading cycles at a fixed pressure of 16 kPa. Figure 4g presents the result. Throughout the entire cycling process, the response signal exhibited no obvious baseline drift or amplitude degradation. Even in magnified views of the initial and final cycles, the response waveforms remained highly consistent, demonstrating excellent long-term mechanical stability and operational reliability that meet the demands of continuous real-world applications. Collectively, these results indicate that the sensor maintains stable electromechanical performance under prolonged cyclic loading, thereby satisfying the reliability requirements for practical long-term applications. In addition, compared with several recently reported flexible pressure sensors [26,27,29,33,34] (Figure 4h), our pressure sensor exhibits excellent linearity, working range, and fatigue cycling stability.

### 3.2. Sensor’s Application for Human Motion Monitoring

**Figure 5:**
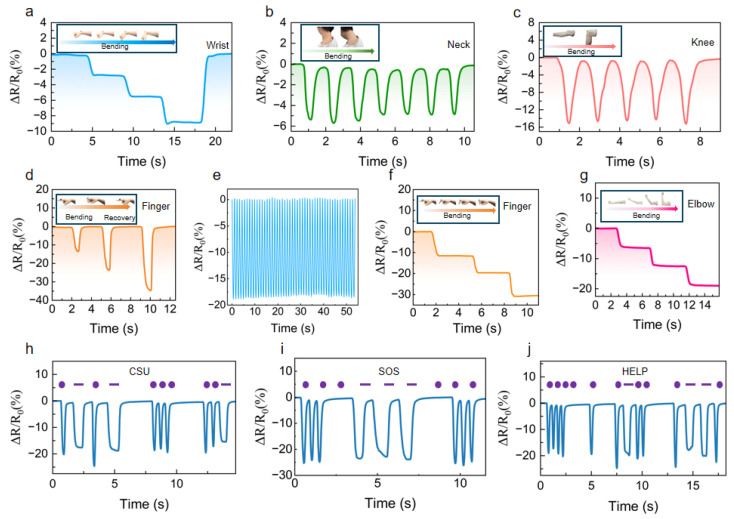
Application of the sensor for monitoring various human physiological motions. (**a**) Relative resistance change during wrist flexion-extension at 30°, 60°, and 90°. (**b**,**c**) Relative resistance change during neck flexion-extension and knee flexion-extension. (**d**) Relative resistance change during finger flexion at 30°, 60°, and 90°. (**e**) Relative resistance change in the sensor under 50 repeated bending cycles. (**f**,**g**) Relative resistance change during finger and elbow flexion at 30°, 60°, and 90°. (**h**–**j**) Application of the pressure sensor in Morse code communication.

First, we attached the sensor to the wrist to monitor its response at different bending angles (30°, 60°, and 90°), and the result is shown in Figure 5a. The results showed that the relative resistance change in the sensor decreased systematically with increasing wrist bending angle, and the signal remained stable at each angle plateau without appreciable relaxation. When the wrist returned to the straight position, the resistance signal quickly recovered to its initial baseline level, demonstrating excellent response stability and recoverability. As shown in Figure 5b,c, we tested the sensor’s response to continuous neck flexion-extension and periodic knee flexion-extension movements. The results indicated that the sensor could capture these periodic motions in real time, outputting clear and repeatable resistance fluctuations in synchrony with joint bending and straightening, thus confirming its capability for monitoring dynamic movements of different body parts. Figure 5d showed that the response amplitude decreased systematically with increasing finger bending angle (30°, 60°, and 90°), with a stable response plateau at each bending stage, reflecting the sensor’s excellent angular resolution and linear response characteristics. In addition, as shown in Figure 5e, we performed a reliability test with 50 repeated bending cycles on the sensor. The results reveal that the relative resistance change in the sensor remains stable during cyclic deformation, without obvious signal drift or performance attenuation. Similarly, when attached to the finger and elbow as illustrated in Figure 5f,g, it produced distinct and distinguishable resistance change signals for flexion at different angles, validating its general applicability for limb joint motion monitoring.

In response to the observation that the sensor exhibits different responses to bending movements and to enable quantitative analysis of joint angles, we have made the following additions. Specifically, we have provided a new table, “Sensor response variation under different joint bending angles”; Table 1 presents the relative resistance change in the sensor corresponding to different bending angles (e.g., 0°, 30°, 60°, 90°) for joints such as the wrist and neck, thereby directly establishing a mapping relationship between joint angle and electrical signal. This addresses the limitation of only qualitatively showing bending/straightening states. Moreover, based on the existing pressure versus relative resistance change calibration curve in Figure 3b (within the range of 0–350 kPa), we further discuss the relationship between the resistance change induced by bending angle and the equivalent pressure/strain: the larger the joint bending angle, the stronger the local compressive strain on the sensor, and the greater the corresponding resistance change. Through Figure 3b, this can be converted into a specific equivalent pressure value, thus providing a basis for quantitative evaluation of different movements. It should be noted that the current angle response table is based on measurements obtained with a single sensor attached to specific locations; different individuals or different attachment positions may introduce some variability. We have pointed out this limitation in the discussion section and have identified systematic calibration of multiple angles and positions, as well as higher-precision angle prediction models, as future research directions.

To further explore the application scenarios of the sensor, we investigated its potential for Morse code communication. By using finger bending actions to simulate the “dot” and “dash” of Morse code, we input three sets of code signals: “CSU”, “SOS”, and “HELP”, and the results are shown in Figure 5h–j. The results showed that the sensor could clearly discriminate between different bending patterns, and the output resistance signal accurately corresponded to the intended long-short sequences, enabling effective information transmission. This demonstrates the sensor’s potential for human–machine interaction and wearable communication.

## 4. Conclusions

In summary, this work develops a bioinspired porous flexible pressure sensor with superior linearity and a broad working range via a stacked “MF-CSP-MF” composite structure. The unique porous framework allows slight elastic deformation under low pressure for precise weak signal detection, while progressive pore collapse and skeleton densification under high pressure alleviate signal saturation and greatly broaden the linear detection region. The sensor achieves an ultra-linear response with R^2^ = 0.999 within 0–350 kPa and maintains stable output over 5000 cyclic tests. High linearity is secured at compression ratios of 20%, 40% and 60%, revealing reliable mechanical and electrical performance under diverse loads. Practical tests confirm its capacity to monitor human joint movements and recognize Morse code, endowing the sensor with promising prospects in electronic skin, flexible sensing and human–machine interaction. Furthermore, the results of this study indicate that the proposed porous-structured sensor exhibits significant potential for achieving a wide sensing range and high-performance sensing. It is expected to find broad applications in fields such as human–machine interaction, dynamic plantar pressure monitoring, and healthcare monitoring. Meanwhile, achieving sensor miniaturization while maintaining a wide range and high linearity performance represents one of the key future research directions for porous-structured sensors.

## Figures and Tables

**Table 1:** Comparison of resistance variation values at different joint bending angles.

Joint	Angle	∆R/R0 (%)
Wrist	30°	−2.8
	60°	−5.5
	90°	−8.8
Finger	30°	−11.5
	60°	−19.6
	90°	−30.6
Elbow	30°	−6.5
	60°	−12.5
	90°	−18.9

## Data Availability

The original contributions presented in the study are included in the article; further inquiries can be directed to the corresponding author.
